# The representativeness of a European multi-center network for influenza-like-illness participatory surveillance

**DOI:** 10.1186/1471-2458-14-984

**Published:** 2014-09-20

**Authors:** Pietro Cantarelli, Marion Debin, Clément Turbelin, Chiara Poletto, Thierry Blanchon, Alessandra Falchi, Thomas Hanslik, Isabelle Bonmarin, Daniel Levy-Bruhl, Alessandra Micheletti, Daniela Paolotti, Alessandro Vespignani, John Edmunds, Ken Eames, Ronald Smallenburg, Carl Koppeschaar, Ana O Franco, Vitor Faustino, AnnaSara Carnahan, Moa Rehn, Vittoria Colizza

**Affiliations:** INSERM, UMR-S 1136, Institut Pierre Louis d’Epidémiologie et de Santé Publique, 27 rue Chaligny, 75012 Paris, France; Sorbonne Universités, UPMC Univ Paris 06, UMR-S 1136, Institut Pierre Louis d’Epidémiologie et de Santé Publique, Paris, France; Università degli Studi di Milano, Milan, Italy; Assistance Publique Hopitaux de Paris, Service de Medecine Interne, Hopital Ambroise Pare, Boulogne Billancourt, France; Department of Infectious Diseases, Institut de Veille Sanitaire (InVS), St Maurice Cedex, 94415 France; Institute for Scientific Interchange (ISI), Turin, Italy; Laboratory for the Modeling of Biological and Socio-technical Systems Northeastern University, Boston, USA; Institute for Quantitative Social Sciences at Harvard University, Cambridge, USA; London School of Hygiene and Tropical Medicine, London, UK; Aquisto-Inter BV, Amsterdam, The Netherlands; Instituto Gulbenkian de Ciȇncia, Oeiras, Portugal; Public Health Agency of Sweden, Stockholm, Sweden

**Keywords:** Influenza, Surveillance, Representativeness, Internet data collection, Participation bias, Selection bias

## Abstract

**Background:**

The Internet is becoming more commonly used as a tool for disease surveillance. Similarly to other surveillance systems and to studies using online data collection, Internet-based surveillance will have biases in participation, affecting the generalizability of the results. Here we quantify the participation biases of Influenzanet, an ongoing European-wide network of Internet-based participatory surveillance systems for influenza-like-illness.

**Methods:**

In 2011/2012 Influenzanet launched a standardized common framework for data collection applied to seven European countries. Influenzanet participants were compared to the general population of the participating countries to assess the representativeness of the sample in terms of a set of demographic, geographic, socio-economic and health indicators.

**Results:**

More than 30,000 European residents registered to the system in the 2011/2012 season, and a subset of 25,481 participants were selected for this study. All age classes (10 years brackets) were represented in the cohort, including under 10 and over 70 years old. The Influenzanet population was not representative of the general population in terms of age distribution, underrepresenting the youngest and oldest age classes. The gender imbalance differed between countries. A counterbalance between gender-specific information-seeking behavior (more prominent in women) and Internet usage (with higher rates in male populations) may be at the origin of this difference. Once adjusted by demographic indicators, a similar propensity to commute was observed for each country, and the same top three transportation modes were used for six countries out of seven. Smokers were underrepresented in the majority of countries, as were individuals with diabetes; the representativeness of asthma prevalence and vaccination coverage for 65+ individuals in two successive seasons (2010/2011 and 2011/2012) varied between countries.

**Conclusions:**

Existing demographic and national datasets allowed the quantification of the participation biases of a large cohort for influenza-like-illness surveillance in the general population. Significant differences were found between Influenzanet participants and the general population. The quantified biases need to be taken into account in the analysis of Influenzanet epidemiological studies and provide indications on populations groups that should be targeted in recruitment efforts.

**Electronic supplementary material:**

The online version of this article (doi:10.1186/1471-2458-14-984) contains supplementary material, which is available to authorized users.

## Background

Monitoring influenza epidemics through surveillance is essential for providing public health recommendations in areas including vaccines, antiviral susceptibility and risk assessment [1]. At the national level, general practice (GP) sentinel surveillance schemes collate information on influenza-like-illness (ILI) of visited patients and, in some cases, collect respiratory specimens.

Alongside these well-established schemes, novel opportunities for surveillance in the general population have been opened by the advent of new technologies that promote the participation of individuals through the Internet, creating information in a bottom-up fashion outside of established practices and routines [2]. A participatory system was introduced in The Netherlands in 2003 for ILI surveillance in the general population by means of an online platform [3], offering a source of disease information generated directly by the users. The system has expanded to other European countries establishing an international participatory surveillance network (Influenzanet). The network has a standardized common framework for data collection [4, 5], thus overcoming possible fragmentations in case definitions and systems design of GP surveillance across countries.

To be of value in providing information to guide health policy, the collected data need to be related to the epidemic situation in the underlying population. In agreement with recommendations for GP surveillance networks [6], here we evaluate the quality of the collected data by assessing the representativeness of the participating (i.e. monitored) individuals in the Influenzanet cohort. The advantage with respect to other surveillance schemes (e.g. GPs or other digital approaches of unsupervised nature, such as web search records [7, 8], online news [9, 10], or tweets [11]) is the ability to ask users about themselves– including geographic, demographic, mobility, socio-economic and health indicator questions; this information can be compared with national statistics. The aim is to identify possible biases to be taken into account for epidemiological analyses. Furthermore, the comparison of representativeness results across countries may guide informed strategies to improve coverage and participation of underrepresented population groups in the following seasons.

## Methods

### Study design

Influenzanet is a European multicenter network [4] for ILI surveillance in the general population through online systems. Starting the 2011/2012 season, Influenzanet was launched with a uniform and standardized data collection approach in seven European countries (The Netherlands [3, 12], Belgium (Flemish region only) [12, 13], Portugal [14, 15], Italy [16], United Kingdom (UK) [17, 18], Sweden [19], France [20, 21]), leveraging on pre-existing participatory surveillance activities [5]. In each country, this surveillance system is coordinated by local research and public health teams and Institutions (see the Additional file 1 for further details).

Focusing on the 2011/2012 Influenzanet season, we analyzed seven national data collection campaigns that started in November 2011 and ended in April or May 2012, with few exceptions (Additional file 1: Table S1). Differences were mainly related to country-specific practical issues (e.g. launch following the Ethical approval in France, or to coincide with public health events or communications for the upcoming influenza season).

Influenzanet consists of a website with centralized information on the network and results from each participating country [4] that links to the national online platforms, each in the national language and with a country-specific name, but characterized by a common website template. National platforms are used to register participants, to give them access to their account where they can upload information, and to publish summary surveillance results in real time.

Participation is voluntary and anonymous, and open to all residents of the countries composing the multi-center network (in France, overseas territories and French individuals under 18 years old were not considered, the latter due to regulatory constraints applied to the first season only). Recruitment occurred with the help of press releases of the supporting institutions, media communications, specific advertising events (e.g. schools activities or science fairs), and through emails and word of mouth. More details can be found on the national platforms [12, 14, 16, 18–20]. In some countries, weekly reports on Influenzanet results were also published within the official national surveillance bulletins [22, 23].

For sensitivity analysis, we also performed the same analyses on the two following influenza seasons, 2012/2013 and 2013/2014.

### Privacy and ethical approval

This study was conducted in agreement with country-specific regulations on privacy and data collection and treatment. Informed consent was obtained from all participants enabling the collection, storage, and treatment of data, and their publication in anonymized, processed, and aggregated forms for scientific purposes. In addition, approvals by Ethical Review Boards or Committees were obtained, where needed according to country-specific regulations. In The United Kingdom, the Flusurvey study was approved by the London School of Hygiene and Tropical Medicine Ethics Committee (Application number 5530). In Sweden, the Influensakoll study was approved by the Stockholm Regional Ethical Review Board (Dnr. 2011/387-31/4). In France, the Grippenet.fr study was approved by the Comité consultatif sur le traitement de l’information en matière de recherche (CCTIRS, Advisory committee on information processing for research, authorization 11.565) and by the Commission Nationale de l’Informatique et des Libertés (CNIL, French Data Protection Authority, authorization DR-2012-024). In Portugal, the Gripenet project was approved by the National Data Protection Committee and also by the Ethics Committee of the Instituto Gulbenkian de Ciência.

### Data collection

To join the network, users registered on their national platform. Upon registration, the user was asked to complete an intake survey, covering demographic factors (age, gender), geographic factors (location of home and work/school expressed at the municipality or zipcode level), socio-economic factors (household size and composition, occupation, educational level, daily transportation means), and health-related factors (including vaccination status against influenza in the 2011/2012 and previous season, diet, pregnancy status, smoking habits, and medical conditions associated with higher risk of influenza complications). The intake survey was standardized and translated whilst preserving the same type and content of questions and possible answers, as well as the same order of questions within the survey, and accounting for the differences related to specific national standards (e.g. schooling structure and associated age/degrees). A few additional questions were added by some platforms due to differences in national public health regulations or to gather additional profiling information. The survey is available in English in the Additional file 2.

A multi-user account was also available to allow the registration of multiple individuals through a single account. The aim was to facilitate group participation (e.g. family members) and also to access groups who otherwise would be unlikely to participate (e.g. children or elderly not familiar with the Internet).

All users were asked to fill in the intake survey at least once, prior to participating to the surveillance. The intake survey could be updated throughout the season (e.g. because of change of residence, vaccination or pregnancy status). When multiple intake surveys were available for a user, in the present study we used the most recently completed one. In the sensitivity analysis, we quantified the type of changes made in the updated surveys and tested the effect of discarding the updates.

**Figure 1:**
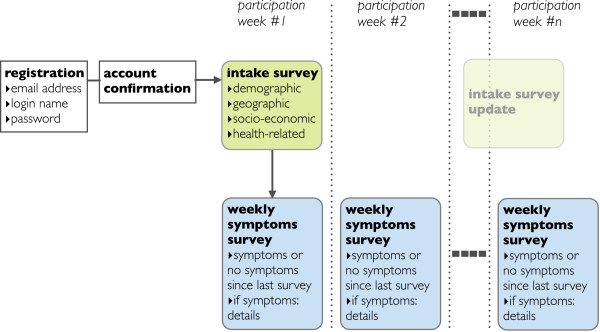
**Flow chart of Influenzanet data collection.** The schematic diagram illustrates the processes of registration, account confirmation, and data collection through intake and weekly symptoms surveys.

Influenza-like-illness surveillance data were obtained through weekly symptoms surveys. No data from the weekly symptoms surveys was considered in this study; however the number and frequency of reporting by each user was used to evaluate the user’s active participation in the surveillance network.A schematic representation of the Influenzanet data collection is shown in Figure 1.

### Inclusion criteria

All intake questionnaires filled in between the start date and the closure date of the data collection campaign for the 2011/2012 season were considered in the analysis. Following previous work [13, 15, 21, 24], we included in our sample only active participants (defined as those who completed an intake survey and at least three weekly symptoms surveys, to avoid results being skewed by sporadic participation). We will refer to these as Influenzanet active participants or Influenzanet participants. We tested different inclusion criteria and performed a sensitivity analysis with the stricter inclusion criterion that each participant filled in at least one weekly symptoms survey per calendar month.

Users who did not specify age/gender details were additionally removed from the sample, as demographic biases could not be assessed nor accounted for in a sample weighting procedure.

### Census and health data sources

We collected national data from a number of socio-demographic datasets and health datasets for all participating countries. In absence of data for the years 2011 or 2012, we relied on the most recent available sources.

Demographic and geographic data were taken from the European Commission portal for European Statistics [25] and from national institutes of statistics. Georeferenced census data were obtained from the Nomenclature of Territorial Units for Statistics (NUTS), a standard geocode for referencing the subdivisions of countries for statistical purposes, developed by the European Union [26]. We considered the NUTS2 level, corresponding to basic regions for the application of regional policies.

All other socio-economic data were taken from European Statistics and national sources: household size and composition [27, 28]; education data [29–31]; employment data [32]; transport habits [33]; vaccination coverage data [34–41]; diabetes prevalence data [42–48]; asthma prevalence data [44, 49–53]; smoking prevalence data [54]; body mass index (BMI) data for France [55].

Commuting data was collected for all countries from national institutes of statistics or departments of transportation [56]. Namely, we used data on the number of daily commuters from location of origin to location of destination.

### Data analysis

The representativeness of the Influenzanet population was assessed through the comparison of its characteristics with those of the general population for each country. We used χ^2^-test for non-continuous sociodemographic variables, and Student’s *t*-test for mean comparisons. All comparisons used 2-tailed tests and a 5% cutoff point. To assess whether differences in participation rates between countries were associated with differences in Internet coverage (access and usage [57]), a test for association between paired samples was considered, using Pearson’s product moment correlation coefficient, Kendall’s τ or Spearman’s ρ. Statistical analyses were performed using the R software version 2.13.2 (R Development Core Team, R Foundation for Statistical Computing, Vienna, Austria, http://www.r-project.org).

Age data were analyzed in 10-years age categories up to an aggregated 70+ class. For France we had a category of 18-19 years old individuals, because of the absence of younger participants during the data collection campaign here analyzed. We additionally split the 60-69 class into two categories, 60-64 and 65-69 years of age, to account for the age definition (65+) of individuals at risk for developing flu-related complications.

Georeferenced data from Influenzanet were mapped from zip codes or municipality resolution to NUTS2 level for comparison with national data. Apart from the geographic and demographic characteristics, all other variables were adjusted by age (10-years categories) and gender.

The household composition question offered a list of age groups to be ticked, next to open fields where to indicate the number of individuals in the household for each selected age group (Intake Q6 in Additional file 2). When no number was indicated, we assumed that one individual belonged to the selected age group.

Commuting data, extracted from countries’ census and from Influenzanet population, were mapped to NUTS2 level. Data were analyzed in terms of networks of nodes and links [58, 59], with nodes representing the NUTS2 regions and directed links the commuting movement between regions. A weight *w*_*OD*_ was also assigned to each link from origin O to destination D to indicate the number of commuters on that connection. Adjusted analyses by geographic distribution of the population were performed (Additional file 1). We assessed whether the Influenzanet links reproduce the *backbone* of the census commuting network defined by extracting for each country a portion of census network of the same size of the Influenzanet commuting network containing the highest traffic links. An alternative definition of backbone was tested for sensitivity analysis using the disparity filter algorithm [60] (Additional file 1). We quantified the overlap between the Influenzanet commuting network and the census one through the Jaccard index, measuring the ratio between the number of common links in the two networks and the total number of links. The index is defined in the range [0,1] where 0 indicates that no common link is observed and 1 indicates that the two sets are identical. We calculated the probability of occurrence of the directed links in the Influenzanet commuting (*P*_*OD*_), given the probability of commuting from O to D computed from national census data and the sample of the Influenzanet participants in region O. Details on the computation are reported in the Additional file 1.

## Results

### Descriptive analysis

A total of 31,674 residents in 7 European countries participated in the 2011/2012 season (Table 1), during a time period of at least 14 weeks. Based on the inclusion criteria, we analyzed a set of 25,481 active participants, representing 80% of the total. Active participation was observed for the majority of individuals in each national sample (from 55% in Italy to 90% in Belgium), with large variations in the active participation rate per country, ranging from 2.1 per 100,000 in Italy to 76.2 per 100,000 in The Netherlands. When compared to Internet access and usage statistics for 2011 (Table 2), we found a positive correlation with the indicators representing access in households (generic Internet access and Internet broadband access) and frequent Internet usage (at least once a week), and a negative correlation with the percentage of individuals who never used the Internet, although all statistical tests were non-significant.

**Table 1:** **Participation to Influenzanet in the 2011/2012 season**

Influenzanet country	No. registered individuals	No. active** participants	% active in sample	No. active in country (per 100,000)
BE	4,362	3,915	90%	56.7
FR*	3,936	3,044	77%	6.2
IT	2,283	1,266	55%	2.1
NL	14,479	12,699	88%	76.2
PT	1,410	1,075	76%	10.2
SE*	2,657	1,676	63%	17.8
UK	2,547	1,806	71%	2.9
Influenzanet	31,674	25,481	80%	8.0

*first season.

**an active participant is defined as having filled at least three weekly symptoms surveys; it is also referred in the article simply as participant (see main text).

**Table 2:** **Participation rates to Influenzanet per country compared to 2011 Internet access and usage statistics**

Country	No. Influenzanet participants per 100,000 (rank)	% individuals using the internet at least once a week (rank)	% internet access in households (rank)	% broadband internet connections in households (rank)	% individuals who never used the Internet (rank)
NL	76.2 (1)	90% (2)	94% (1)	83% (2)	7% (2)
BE	56.7 (2)	78% (4)	77% (4)	74% (4)	14% (4)
SE	17.8 (3)	91% (1)	91% (2)	86% (1)	5% (1)
PT	10.2 (4)	51% (7)	58% (7)	57% (6)	41% (7)
FR	6.2 (5)	74% (5)	76% (5)	70% (5)	18% (5)
UK	2.9 (6)	81% (3)	85% (3)	83% (2)	11% (3)
IT	2.1 (7)	51% (6)	62% (6)	52% (7)	39% (6)

Among the sample of active participants, 83% had a single membership account (variation from 69% for Italy to 89% for Belgium), 9% belonged to a multiple account with 2 active participants (from 7% for Belgium to 12% for the UK), and 8% belonged to an account with 3 or more participants.

Overall, 89.1% of participants never updated their intake survey (variation from 78.7% for Italy to 93.5% for Sweden), 8.8% updated it twice, and 2.1% updated it at least three times.

### Geographic and demographic characteristics

**Figure 2:**
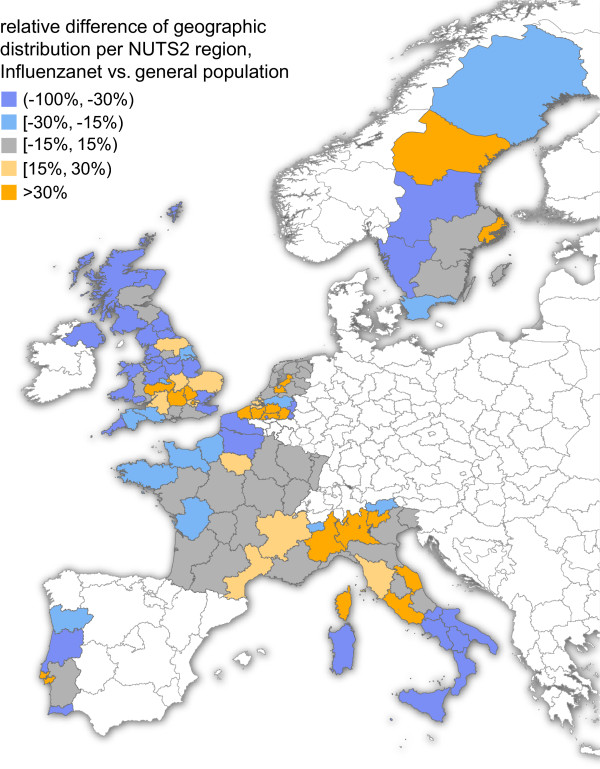
**Geographic distribution of Influenzanet participants at the level of NUTS2 regions.** The color code indicates the relative difference between the geographic distribution of Influenzanet population and the corresponding general population data. The map was created with the collected data using ArcGIS Software and publicly available geographic datasets [25].

All 113 NUTS2 regions of the countries analyzed were covered by the study, with an active participation rate per region varying between 0.3 per 100,000 (Calabria region, Italy) and 96.1 per 100,000 (Utrecht region, The Netherlands). Geographic repartitions of Influenzanet participants per region were statistically different from census data (Additional file 1: Figure S3). Two countries – France and The Netherlands – reported a majority of regions (12 out of 22 in France, and 8 out of 12 in The Netherlands) having a relative difference between Influenzanet population and national population in the range [-15%,15%) (Figure 2). Out of the total of 113 NUTS2 regions, 34 (30%) had a relative difference in the range [-15%,15%), distributed differently across countries (12 regions in France, i.e. 35.3% of the 34 regions in this range; 8 (23.5%) in The Netherlands; 6 (17.7%) in Italy; 5 (14.7%) in the United Kingdom; 2 (5.9%) in Sweden; and 1 (2.9%) in Portugal).

**Figure 3:**
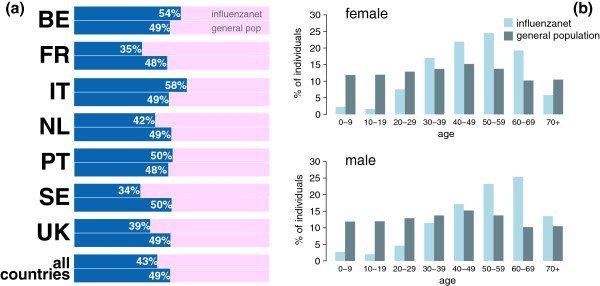
**Gender and age profiles of Influenzanet population and comparison with the general population.** Gender repartition is displayed for each country and aggregated for all countries **(a)**; age profile in 10-years classes per gender is shown aggregated for all countries (country level statistics are reported in Additional file 1: Figure S4) **(b)**.

Regarding the gender distribution in the Influenzanet population, the countries are split into three different sets: i) *male-prevalent* countries with a larger proportion of males participating in the project compared to the national population distribution (Belgium, Italy; *p* < 10^-4^); ii) *female-prevalent* countries (The Netherlands, United Kingdom, Sweden, and France; *p* < 10^-4^); iii) a statistically representative population by gender (Portugal, *p* = 0.08) (Figure 3a). If we consider the aggregated data across all 7 countries Influenzanet participants are more likely than the general population to be female (56.8% vs. 50.9%, *p* < 10^–4^).

**Figure 4:**
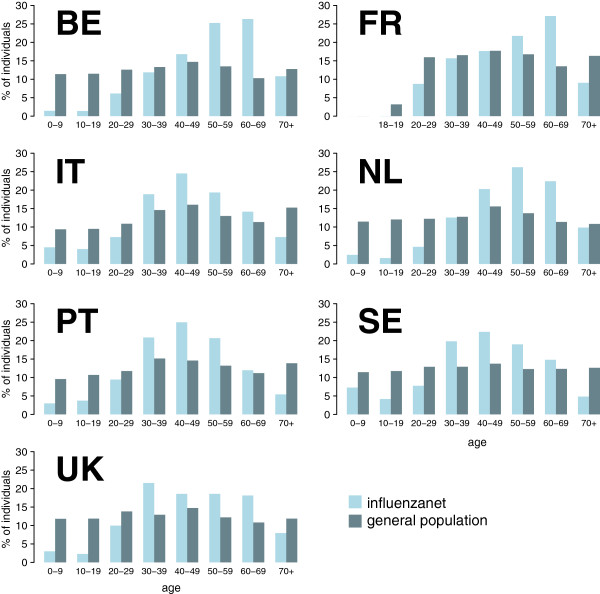
**Age profile of Influenzanet participants and comparison with the general population per country.** Age distribution is shown in 10-years age classes. Country profiles by age and gender are reported in Additional file 1: Figure S4.

Participants were found to be older than the general population (*p* = 10^–3^ for Italy, *p* < 10^–5^ for all other countries), except the female participants in Portugal who were statistically representative of the country’s female population in terms of age (*p* = 0.5), and in Italy who were younger than the corresponding census group (*p* = 0.01, Table 3). Overall, there was an overrepresentation of the adult classes ([40-69]y) and an underrepresentation of the youngest classes ([0-29]y). The latter results are obtained for the entire Influenzanet population and for both genders (Figure 3b), and they are also valid at country level, except for France in the [40-49]y class (Figure 4, with no breakdown by gender). Overrepresentation of the [60-69]y class was confirmed by further breaking down the age group, below and above 65 years of age (except for the [65-69]y class in Portugal that is found to be representative of the corresponding age class in the general population, Additional file 1: Table S2).

**Table 3:** **Average age of Influenzanet participants and comparison with the national statistics (all** ***p*** **< 10** ^**–5**^ , except * ***p*** **= 0.5, †0.01** **<** ***p*** **<** **0.03, ††0.001** **<** ***p*** **<** **0.006)**

Gender	Influenzanet country	Influenzanet	General population
		Years (95% CI)	Years
All	BE	52.8 (52.3 – 53.3)	42.0
	FR	52.0 (51.5 – 52.5)	48.6
	IT††	45.9 (45.0 – 46.9)	44.3
	NL	51.6 (51.3 – 51.9)	40.8
	PT	44.9 (44.0 – 45.9)	39.7
	SE	43.7 (42.8 – 44.5)	41.7
	UK	47.0 (46.2 – 47.8)	40.5
Female	BE	49.0 (48.3 – 49.7)	43.3
	FR	50.8 (50.2 – 51.4)	41.2
	IT†	43.7 (42.3 - 45.2)	45.6
	NL	49.7 (49.3 – 50.0)	41.6
	PT*	42.4 (41.0 – 43.8)	41.9
	SE††	44.3 (43.3 – 45.3)	42.9
	UK	45.5 (44.6 – 46.5)	41.5
Male	BE	56.0 (55.4 – 56.7)	40.7
	FR	54.3 (53.4 – 55.2)	38.2
	IT	47.5 (46.3 – 48.8)	43.0
	NL	54.3 (53.9 – 54.8)	40.0
	PT	47.5 (46.1 – 48.9)	37.6
	SE†	42.5 (40.8 – 44.2)	40.6
	UK	49.4 (48.0 – 50.7)	39.4

The class of young adults, from 30 to 39 years of age, showed different results depending on gender (Figure 3, when all countries are considered) and on the country (Figure 4 and Additional file 1: Figure S4).

Gender-specific differences in the representativeness of Influenzanet participants are also found in the older classes. Each country reported an underrepresentation of the 70+ class when all participants are considered, with the male class being however overrepresented in the majority of countries (Belgium, France, The Netherlands and UK, Additional file 1: Figure S4). This gender disproportion is also confirmed if we consider all Influenzanet countries aggregated (Figure 3b).

### Mobility features

Among the active participants, 55% (13,748 individuals) provided information on their school/work locations. The majority of participants reported commuting within the administrative region of their residence. The ratio between across-regions and within-regions commuters varied from 48% (UK) to 2.5% (Italy), and was statistically representative of the corresponding census ratios (Table 4, *p* ≥ 0.1 for all countries).

**Table 4:** **Average ratio between the number of individuals commuting outside and within their region of residence (all** ***p*** **>** **0.1) and comparison with national statistics**

Influenzanet country	Influenzanet	General population
	Ratio between across-regions and within-regions commuters (95% CI)	Ratio between across-regions and within-regions commuters (95% CI)
BE	0.429 (0.021 - 1.000)	0.371
FR	0.053 (0.000 - 0.213)	0.037
IT	0.025 (0.000 - 0.135)	0.014
NL	0.189 (0.102 - 0.343)	0.182
PT	0.164 (0.000 - 0.806)	0.041
SE	0.028 (0.0 - 0.102)	0.051
UK	0.478 (0.0 - 2.746)	0.251

**Figure 5:**
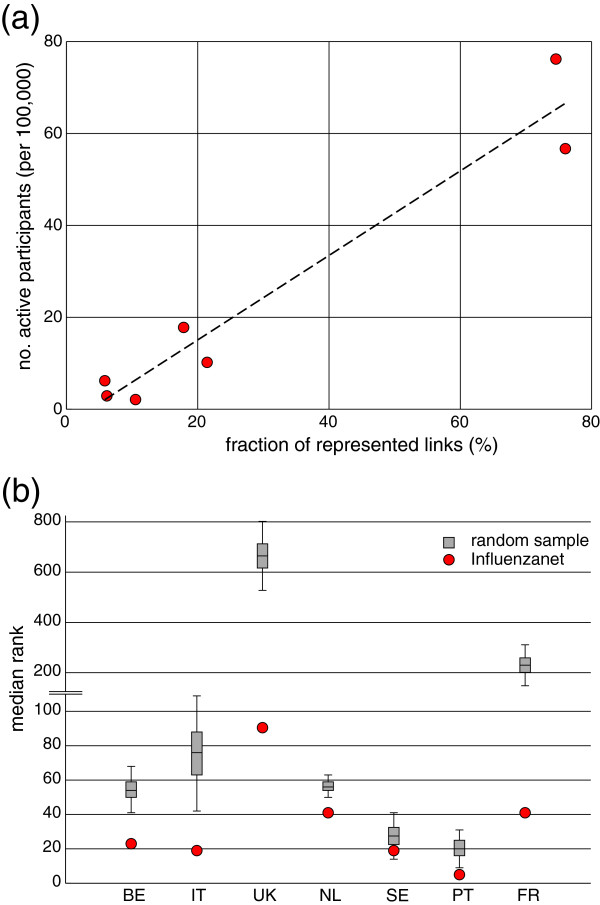
**Quantitative analysis of the Influenzanet commuting network. (a)** Linear correlation between the fraction of commuting links represented in Influenzanet and the fraction of active participants per country (*R* ^2^ =0.96). **(b)** Statistical analysis of the traffic weights of the links represented in Influenzanet. For each country, the median rank of the commuting links represented in the Influenzanet population (red dot) is compared with a random sample (grey bar). Commuting links are ranked for decreasing probability of occurrence *P*_*OD*_. Median ranks are smaller than the corresponding value for the random sample, and outside of the confidence interval for all countries except Sweden.

**Figure 6:**
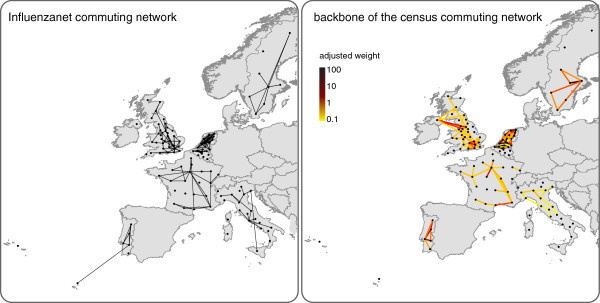
**Comparison between the Influenzanet commuting network (left) and the backbone of the census commuting network (right).** The color code associated to the links in the census commuting network is proportional to the adjusted weight (from yellow to dark-red). Both networks are directed, arrows are omitted for the sake of visualization. Maps were created with the collected data using ArcGIS Software and publicly available geographic datasets [25].

In the census commuting network all NUTS2 regions have either in-coming or out-going commuting links with other regions in the country. In the Influenzanet network, only a portion of links were represented (Additional file 1: Table S3) with several regions remaining disconnected in the network, as they did not report either incoming or outgoing commuters (4 regions in France, 3 in Italy, 2 in Portugal, 1 in Sweden and 3 in the UK). The fraction of represented links correlated well with the participation rate in the country (Figure 5a). Moreover, represented links were in general found among the ones with higher probability *P*_*OD*_ of occurrence (Figure 5b).The Influenzanet commuting network was able to capture some of the relevant features of the census commuting patterns (Figure 6). Where a small fraction of links was represented, Influenzanet commuting network was still able to reproduce the strongly connected portions of the census commuting network in given regions (for instance, in the North of Italy, in the South of France, and in the South of Sweden). Commuting flows to/from central urban areas, like Paris and London, and the triangular pattern in the North of Portugal were also recognizable. Variations were observed in connections to peripheral areas, with some cases being reproduced (Corsica to Metropolitan France, and Northern Ireland to the rest of Great Britain), whereas others being absent from the Influenzanet commuting network (Madeira archipelago to continental Portugal, and North-South axis in Sweden).

Census backbones and Influenzanet networks showed an overlap ranging from 0.18 (Sweden) to 0.85 (Belgium) (Additional file 1: Table S4). The adoption of an alternative definition of network backbone displays a lower similarity between the two networks (Figure A5).

**Figure 7:**
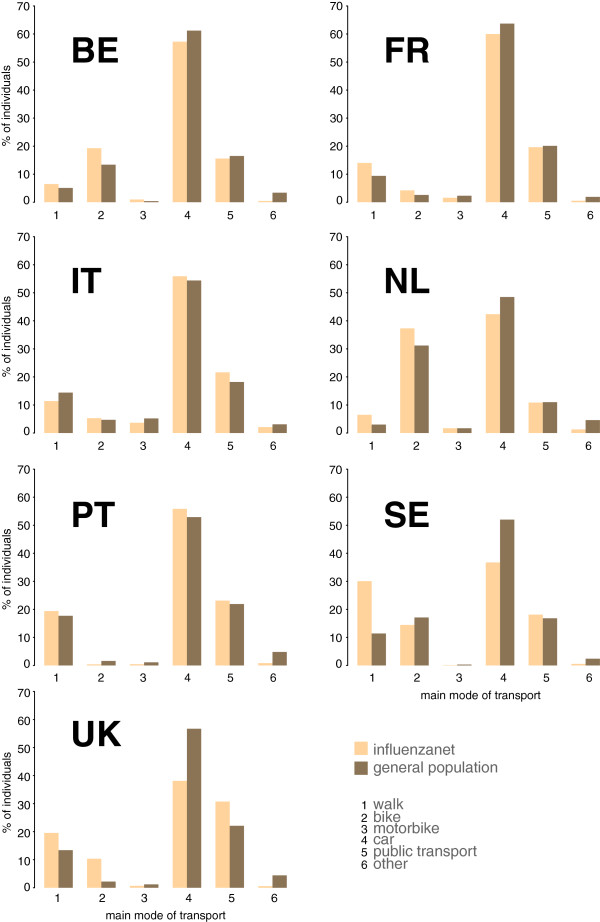
**Distribution of the use of transportation modes for Influenzanet participants and comparison with national statistics.**

The comparative analysis on the mode of transport on a regular day among participants of 15 years or older shows that the main mode of transport was statistically representative of the national data for one country only (Italy, Figure 7). For all other countries, differences in the distributions were found to be significant (*p* < 10^–4^).

### Socio-economic factors

**Figure 8:**
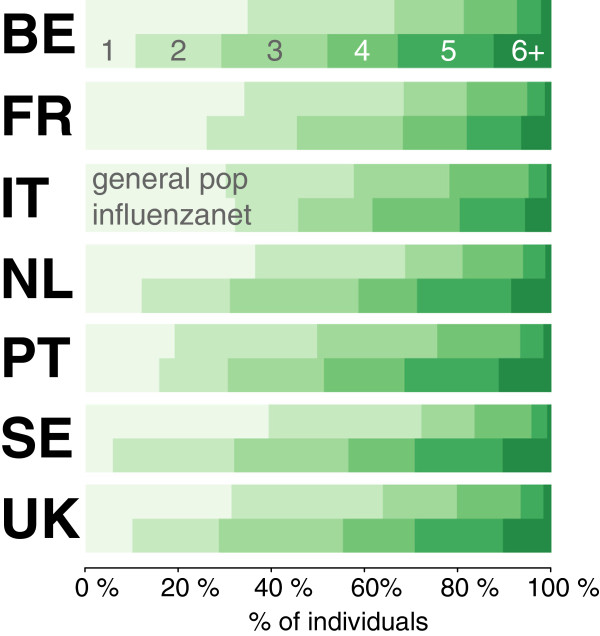
**Household size distribution for Influenzanet participants and comparison with national statistics.**

Influenzanet participants belonged on average to larger households than the general population (Table 5, *p* < 10^–3^ for each country). The distributions of the number of household’s members of Influenzanet participants were statistically different from the national ones (*p* < 10^–4^, Figure 8). All countries except Italy reported a smaller proportion of households of size equal to 1, with the smallest value observed in Sweden (5.87% vs. 39.3%) and the largest one observed in Italy (32.08% vs. 30.1%).

**Table 5:** **Average household size of Influenzanet participants and comparison with national statistics (all** ***p*** **< 10** ^**–3**^ **)**

Influenzanet country	Influenzanet	General population
	Household size (95% CI)	Household size
BE	3.4 (3.3 – 3.5)	2.3
FR	2.9 (2.8 – 3.0)	2.2
IT	2.8 (2.7 – 3.0)	2.4
NL	3.2 (3.2 – 3.3)	2.2
PT	4.0 (3.2 – 4.8)	2.6
SE	3.9 (3.3 – 4.5)	2.1
UK	4.0 (3.1 – 5.0)	2.3

Country-specific differences were found regarding employment representativeness (Table 6). No significant difference was found in the UK; the employed were marginally oversampled in Portugal and marginally undersampled in Sweden. Larger discrepancies are found in the rest of the countries, with Belgium, Italy, and France overestimating the national employment rates, and The Netherlands showing the opposite trend.

**Table 6:** **Employment rate in the [15-64]y class of age and comparison with national statistics (all** ***p*** **< 10** ^**–3**^ , except * ***p*** **= 0.09, †0.01** **<** ***p*** **< 0.05**

Influenzanet country	Influenzanet	General population
	% (95% CI)	%
BE	68.6 (66.7 – 70.4)	61.9
FR	70.9 (68.8 – 73.0)	63.8
IT	66.2 (61.2 – 71.0)	56.9
NL	72.6 (71.7 – 73.6)	74.9
PT†	68.2 (64.6 – 71.5)	64.2
SE†	71.3 (68.4 – 74.0)	74.1
UK*	68.1 (65.4 – 70.7)	70.4

In the three countries where education data at the general population level was available for comparison with Influenzanet data (France, Portugal and Sweden), participants had a higher education level than the general population (Table 7).

**Table 7:** **Education level of Influenzanet participants and comparison with national statistics (all** ***p*** **< 10** ^**–6**^ **)**

Influenzanet country: indicator	Classes	Influenzanet	General population
		% of individuals	% of individuals
FR: individuals with at least high-school level	[25-34]y (female;male)	95.1; 96.9	70.2; 61.7
	[35-44]y (female;male)	93.6; 94.1	54.9; 47.6
	[45-54]y (female;male)	83.6; 87.1	39.4; 32.9
	[55-64]y (female;male)	81.8; 71.8	30.1; 29.9
PT	No qualification ([15-64]y; 65+) 65+)	0; 0	3.6; 36.2
	GCSE ([15-64]y; 65+)	3.5;10.3	60.2; 55.7
	A-level ([15-64]y; 65+)	16.3;27.0	20.6; 3.0
	Higher ([15-64]y; 65+)	80.2; 62.9	15.6; 5.1
SE: individuals in [20-64] age class	No qualification (female; male)	0; 0	13; 17
	GCSE (female; male)	2; 3	23; 26
	A-level (female; male)	17; 25	23; 25
	Bachelor (female; male)	16;14	16; 14
	Higher (female; male)	66; 57	25; 18

### Health factors and vaccination

**Figure 9:**
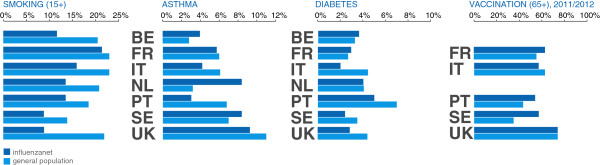
**Prevalence of different health indicators: smoking in the 15+ population, asthma, diabetes, and vaccination against influenza in the 65+ population.** Influenzanet prevalence is compared to national statistics.

The prevalence of daily smokers in the 15+ age class is significantly lower in Influenzanet participants than in the general population across all countries (*p* < 10^–3^) except in France where it is statistically representative (21.51% vs. 23.3%, *p* = 0.08) (Figure 9). Similar results are obtained for the male population, whereas in the female class also Portugal and Italy, in addition to France, report Influenzanet smoking prevalence in agreement with national statistics (Additional file 1: Table S5).

The percentage of Influenzanet participants reporting asthma is significantly lower than in the general population of Portugal (3.04% vs. 6.80%,*p* < 10^–5^), Italy (4.2% vs. 6.1%, *p* < 10^–2^), and the UK (9.2% vs. 11%, *p* = 0.02). The opposite trend is obtained for The Netherlands (8.4% vs. 3.2%, *p* < 10^–6^) and Belgium (3.95% vs. 2.8%, *p* < 10^–4^). No significant difference was found in France (5.9% vs. 6%, *p* = 0.8). Results are reported in Figure 9.

Influenzanet diabetes prevalence was in agreement with national data for three countries (The Netherlands, Belgium, and France, with *p* = 0.8, *p* = 0.2, and *p* = 0.7, respectively), and lower for the others (*p* = 0.02 for Sweden, *p* = 0.01 for Portugal, *p* ≤ 10^–3^ for Italy and UK, Figure 9).

Vaccination coverage against influenza in the 65+ age class during the 2011/2012 season was larger in the Influenzanet participants of France, Portugal and Sweden, whereas it was statistically representative in Italy (57.2% vs. 62.7%, *p* = 0.8) and UK (74.21% vs. 74%, *p* = 0.98) (Figure 9). In the 2010/2011 season, vaccination coverage was higher among Influenzanet participants in all countries (*p* < 10^–4^), except in Italy where vaccinated 65+ individuals were strongly underrepresented (35% vs. 62%, *p* < 10^–4^), and in UK where vaccination coverage was in agreement with national data (75% vs. 73%, *p* =0.55, see Additional file 1: Table S6). Dutch data were not available for comparison for the 2011/2012 season and Belgian data were not available for both seasons.

### Sensitivity analysis

Repeating the analysis with a stricter inclusion criterion produced no qualitative differences in the results presented.

The updates of the intake survey for 10.9% of the total number of participants most frequently concerned the participant’s job (place of work; occupation; main activity), her weight (in the French survey only, where a question on weight and height was added to evaluate the participant’s BMI), her mean of transport (main mean of transport; time spent daily on public transportation), and her place of living. These changes do not affect the results obtained for the representativeness in terms of age, gender, household, and health indicators. The changes in the geographic and job indicators produced no qualitative differences in the results presented.

The representativeness analysis on the following two seasons (2012/2013 and 2013/2014) showed that the obtained results are robust in time (Additional file 1). No qualitative difference was observed, except for the Influenzanet vaccination coverage in France that was found to be representative of the corresponding value in the general population, differently from the 2011/2012 season. Differences in the participation of specific age groups were observed in some countries (e.g. in the UK where a higher participation of school-aged children was reported in the 2013/2014 season thanks to school-specific activities and communication campaigns), without altering the overall picture of lack of representativeness in terms of age observed for all participating countries.

## Discussion

31,674 residents in 7 European countries joined the online surveillance study in the first season (2011/2012) where a standardized and uniform data collection approach was adopted by the Influenzanet Consortium. Active participation was observed for 80% of the participants and covered all NUTS2 regions included in the project. Participation varied widely across countries, geographic regions, gender groups, and age classes. This is most likely related to different factors, namely: the reachability of a given portion of the population obtained through communication campaigns; the availability, usage of and familiarity with the Internet (which is used in this study as the mean to collect data); and the self-selection of participants, or ‘volunteer effect’, and the underlying interest towards the object of the study [61].

Results seem to indicate that coverage biases due to the Internet may partly explain the observed variability in participation per country, however all tests were non-significant likely due to the small number of data points. Belgium and Portugal showed a better ranking in participation rates with respect to the various Internet indicators, pointing to a larger participation than expected based on country ranking for Internet usage only, which is likely due to the longer history of the national platforms (Belgium from 2003 together with The Netherlands, and Portugal from 2005). The Netherlands, France, and Italy ranked in participation approximately as expected by Internet access and usage statistics. Conversely, Sweden and the United Kingdom were ranked lower in participation rates (3^rd^ and 6^th^, respectively) than according to Internet statistics. It is important to note that for France and Sweden it was their first season in the project.

Geographic distribution within each country was not representative, and a larger participation was generally observed in those regions hosting the laboratory/Institution conducting the study, likely reflecting a more powerful effect of communication campaigns at the local level. Other initiatives, geographically limited, appear to be responsible for large participation rates in the population. This is for example the case of the Corsica region, with a participation rate of 3.5 per 100,000 vs. 2.4 per 100,000 observed in the region of the Ile de France (hosting the Supporting Institution), following the diffusion of localized communication campaigns and Influenzanet activities at schools in the region supported by a regional project [62].

An unbalance in the participation by gender was observed, except in the case of Portugal. Two opposed aspects may be at play in the gender imbalance. On one hand, previous studies suggest that women are on average more interested in health-related topics and also exhibit a more active information-seeking behavior [63, 64]. Such gender-specific behavior may thus lead to a more likely voluntary female participation in a health-related project like Influenzanet, as observed in The Netherlands, UK, Sweden, and France. Results showing a higher tendency of participation of larger households in the study may further support this hypothesis, as possibly driven by women’s interest in family and children care [65].

On the contrary, Belgium and Italy showed a larger fraction of male participants with respect to the national partition by gender. This might be explained by another gender-specific aspect, regarding the usage of and familiarity with technology in general. Internet usage differs by gender across all countries, with a larger fraction of men accessing the Internet at least once per week compared to women [57]. Interestingly, the countries with the largest relative difference in the gender-specific access to the Internet (Italy, Portugal and Belgium, with a relative difference of 18%, 11%, and 7%, respectively) were also the countries with a larger prevalence of male Influenzanet participants (Belgium and Italy) or displaying a representative population by gender (Portugal). A larger disproportion in men’s vs. women’s Internet access appears therefore to balance out the female volunteering effect due to health-interest, family care, and information-seeking behavior.

The Influenzanet population was not representative in terms of age, with an overrepresentation of the [40-69]y class (for each gender), an underrepresentation of the younger age classes, [0-29] (for each gender), and of the elderly (age ≥ 70y, for all countries when both genders are considered together). Internet usage statistics report a decreasing dependence on age [57], with larger (e.g. Italy and Portugal) to smaller (e.g. The Netherlands and Sweden) variations by age classes. This decreasing rate by age may explain the low rates of participation observed in the 70+. To achieve a better representativeness of individuals in this class, the surveillance system will need to design targeted communication campaigns for this group and, most importantly, facilitate the accessibility to the project. It is interesting to note that individuals in the [60-69] age class are largely overrepresented. We tested whether this may be induced by a specific interest and concern of individuals of 65+ years of age for whom influenza vaccination is recommended in Europe, but found no major difference in the representativeness of [60-64] vs. [65-69] class to support this hypothesis.

Underrepresentation in the [0-9] and [10-19] classes of age may be due to the impossibility to access the Internet in an unsupervised way for the youngest children, and to the unlikelihood of being exposed to the project for the older ones. The system already incorporates the possibility of adding multiple users to an account managed by a single participant who is supposed to facilitate the input of data for individuals who cannot or are not familiar with Internet tools. The results of this study for the 2011/2012 season indicate, however, that more specific efforts in reaching out to younger age classes are needed, for instance through projects and communication/entertainment actions at schools. Such actions may be for example responsible for the increase in participation rates in school-aged children observed in the UK in the 2013/2014 season.

A lack of interest in influenza or health-related topics may be at the basis of the underrepresentation of the [20-29] age class, since this is the group having the most largely diffused usage of new technologies, with an at least weekly Internet access reported for more than 88% of individuals between 16 and 34 years old for all countries studied, with the exception of Italy (81% for [16-24]y and 70% for [25-34]y) and Portugal (89% and 77%, respectively). The class of [30-39] years old instead showed a different participation behavior depending on the gender (overrepresentation of females and underrepresentation of males) and on the country (underrepresentation in Belgium, France and The Netherlands, when both genders are considered, opposite trend elsewhere). This age class may represent the transition between young-specific lack of interest for the project and the raise of family-specific interest for health-related information. The average age at first childbirth is indeed found between 28 years (Belgium) and 30 years (Italy) in 2010 [66]. Other possible mechanisms may clearly come into play, such as e.g. a more general increased responsibility towards society and public good.

Once the non-representative nature of the Influenzanet population in terms of age and gender was adjusted for, commuting patterns registered by Influenzanet reproduced well the ratio between the within-region and the across-region number of commuters, recovering a feature that is relevant for the spatial spread of influenza. The proportion of census links represented in the Influenzanet network was larger for the countries with higher number of active participants, showing that a better representativeness of the topology of the network can be reached with higher levels of participation. When only a small fraction of links was represented, those were in general the ones with higher census traffic, i.e. the network backbone.

The analysis of transportation modes showed that the Influenzanet sample, despite being non representative for 6 countries out of 7, reproduced some of the aspects of the general population transport behavior, like the top three transportation modes, that were the same in the Influenzanet and in the general population for all countries except Sweden.

Participants in general had higher education levels compared to the general population, which is in agreement with previous studies employing web-based surveys [67, 68], and is likely induced by the non-representative nature of Internet users (Internet usage dramatically increases with education level [57]) and of the sample of individuals highly engaged in the survey’s topic.

Our interpretation of partially incomplete data for the household composition (see Methods) offers a lower boundary for the household size, therefore it does not qualitatively alter our findings on larger household sizes found for Influenzanet participants. Other assumptions adopted for the study were tested for sensitivity (i.e. stricter inclusion criteria and consideration of the first intake only neglecting following updates) and no qualitative differences were observed in the results.

The Influenzanet sample contained fewer smokers than expected from national statistics, with few exceptions (representativeness for France for both genders and for Portugal and Italy for the female sample only). International comparability on such statistics is however limited due to the lack of standardization in the measurements of smoking habits in health interview surveys across EU member states (see e.g. differences found across different sources, Refs. [54] and [21]). For example, there are variations in the wording of questions and in the response categories used in surveys for smoking behaviors (e.g. smoking daily vs. regularly). Our results consider the Influenzanet responses for daily smoking habits (i.e. less than 10, 10 or more cigarettes per day, excluding occasional smokers) compared to the national statistics defined in terms of ‘daily smoking’ [54].

Vaccination coverage against influenza in the 65+ age class was statistically representative of national coverage in Italy and the UK, and it was higher in the samples of the other countries. Vaccination coverage reported for Italy for the previous season (participants were asked about their vaccination status in the previous season too) was much smaller than what has been declared for the season under study (the latter being also statistically representative of national data). No clear explanation is available, given that the sample of individuals declaring the vaccination status is the same. It may be due either to memory biases in the reporting of previous season vaccination behavior, or to change of vaccination behavior from one season to another. The 2010/2011 season was indeed the first influenza season following the 2009 H1N1 influenza pandemic, and the reported coverage may be the result of the negative impact of the controversies related to the pandemic vaccination campaign of 2009/2010 on subsequent seasonal influenza vaccination coverage. While this hypothesis was explicitly tested in some countries where no association was found [69], we are not aware of similar studies being conducted in Italy, and we argue that the large variability observed in the attitudes towards vaccination uptake during the H1N1 pandemic [70] may possibly lead to different results that are country-specific.

Overall, health-related results further indicate a tendency of Influenzanet participants towards better health and towards health care, with few exceptions. Furthermore, an analysis on the Body Mass Index of French participants have shown that they were less frequently found to be overweight and obese than the French population [21], further supporting such tendency.

For sensitivity analyses we also tested the robustness of our findings for the two influenza seasons following the standardization of the Influenzanet platform. The only qualitative difference was found in the vaccination coverage of the 65+ Influenzanet population in France that was representative of the corresponding national value for both 2012/2013 and 2013/2014 seasons, whereas in the 2011/2012 season a marginal overrepresentation was observed. It is to be noted however that some statistics for the general population for some of the indicators considered here were not available for all countries at the time of the study (e.g. vaccination coverage for the last influenza season, or asthma and diabetes incidences). Other differences, however not altering the findings reached for the 2011/2012 season, included an increase in the participation rate of school-aged children in the UK, following targeted communications in the country. Larger quantitative differences that may alter the conclusions of this study may be found on longer timeframes of data collection, induced by population changes in some of the indicators that may drive the participation to the surveillance scheme. For example, in the longitudinal study of eight seasons of the Belgian platform, Vandendijck and collaborators found a marked increase in participation in the [60-69] age class, likely attributable to the growing internet usage in this age group during that timeframe [13].

In addition to the self-selection bias, another potential limitation of the study is induced by the employed data collection methodology that may have an effect on data reliability when participants self-report inaccurate information. This may happen unconsciously, e.g. due to the fact that participants mistakenly introduce wrong data or might forget to report an information, or as the result of a deliberate action. In the first case, while simple mistakes in completing the surveys may be automatically checked by the system (as e.g. a date of birth in the future) or avoided with design improvements, all errors related to misunderstandings, subjective interpretation or memory effects in the reporting would go undetected. Studies have found that Web participants’ responses contained less random and systematic error than their telephone counterparts [71]. This was explained as an effect of the lack of social compliance towards the interviewer and the availability of a longer time to process the information at the individual’s own pace [72]. Moreover, memory effects leading to a systematic error known as recall bias are expected to occur when surveying participants’ behavior on a large set of indicators regarding events or experiences from the past. We evaluate that such bias is unlikely to occur in the intake survey of Influenzanet, as the questions asked refer to standard demographic information and everyday habits or conditions (e.g. smoking behavior, main mean of transportation, presence of allergies, etc.). For the same reason, also misunderstandings and wrong interpretations of the questions are unlikely to occur.

The only question referring to a particular event in time contained in the Influenzanet intake survey is the one on the vaccination status. If the vaccination occurs after the completion of the intake survey, the participant may forget to update the information on her personal space, thus inducing a bias in our results. We evaluate that such cases, if present, would represent a small fraction of the total, as the Influenzanet surveillance campaign typically starts after the vaccination campaign in each of the countries. Nonetheless, a simple reminder concerning the update of the vaccination status can be easily implemented to overcome this issue.

Concerning deliberate actions of providing inaccurate data on online surveys, the probability of filling in fraudulent data in a web-based survey, though possible, is expected to be very limited due to the absence of specific incentives, and the time resources needed to perform the fraudulent action.

## Conclusions

The analysis of the characteristics of approximately 25,000 participants in the Influenzanet network of online platforms for influenza-like-illness surveillance showed a large variability across countries in terms of representativeness. The youngest and oldest age classes were all underrepresented, and gender representativeness was reached only for one country out of seven. Participants’ households were found to be larger than those of the general population, and participants’ health indicators overall indicated a higher concern for health-related issues.

The advantage of the system is to allow the evaluation of representativeness along a large set of population aspects. The study indicated areas in which specific strategies and updates in future surveillance may be envisioned for the recruitment of undersampled groups of the general population. The evaluation findings will be used to correctly interpret epidemiological data and assess risk factors to inform public health policy.

## Electronic supplementary material

Additional file 1: **The file contains additional information on the Influenzanet system, the methods used in the analysis, and additional results.** (PDF 2 MB)

Additional file 2: **The file contains the Influenzanet intake survey in its English version.** (PDF 158 KB)
