# Supplementary material for: The representativeness of a European multi-center network for influenza-like-illness participatory surveillance
Source: BMC Public Health. 2014 Sep 20;14:984. doi: 10.1186/1471-2458-14-984 (PMC4192744; doi:10.1186/1471-2458-14-984)
Supplement: Supplementary file 1 — Additional file 1: The file contains additional information on the Influenzanet system, the methods used in the analysis, and additional results. (PDF 2 MB) [file 12889_2014_7121_MOESM1_ESM.pdf]

Additional File 1

The representativeness of a European multi-center network for influenza-like-illness participatory surveillance

Pietro Cantarelli[1,2,3], Marion Debin[1,2], Clément Turbelin[1,2], Chiara Poletto[1,2], Thierry Blanchon[1,2], Alessandra Falchi[1,2], Thomas Hanslik[1,2,4], Isabelle Bonmarin[5], Daniel Levy-Bruhl[5], Alessandra Micheletti[3], Daniela Paolotti[6], Alessandro Vespignani[6,7,8], John Edmunds[9], Ken Eames[9], Ronald Smallenburg[10], Carl Koppeschaar[10], Ana O. Franco[11], Vitor Faustino[11], AnnaSara Carnahan[12], Moa Rehn[12], Vittoria Colizza[1,2,6*]

1) INSERM, UMR-S 1136, Institut Pierre Louis d'Epidémiologie et de Santé Publique, Paris, France
2) Sorbonne Universités, UPMC Univ Paris 06, UMR-S 1136, Institut Pierre Louis d'Epidémiologie et de Santé Publique, Paris, France
3) Università degli Studi di Milano, Milan, Italy
4) Assistance Publique Hopitaux de Paris, Service de Medecine Interne, Hopital Ambroise Pare, Boulogne Billancourt, France
5) Department of infectious diseases. Institut de Veille Sanitaire (InVS), St Maurice Cedex, 94415, France
6) Institute for Scientific Interchange (ISI), Turin, Italy
7) Laboratory for the Modeling of Biological and Socio-technical Systems Northeastern University, Boston, USA
8) Institute for Quantitative Social Sciences at Harvard University, Cambridge, USA
9) London School of Hygiene and Tropical Medicine, Great Britain
10) Aquisto-Inter BV, Amsterdam, The Netherlands
11) Instituto Gulbenkian de Ciência, Oeiras, Portugal
12) Public Health Agency of Sweden, Sweden

1. Influenzanet: some details

Originated from a science communication project in 2003 in The Netherlands, Influenzanet was then expanded to other European countries and established in 2011 a standardized common approach for collecting data in the realm of influenza-like-illness surveillance. A detailed history of the evolution of Influenzanet system is provided in Ref. [6].

The system is supported in each country by:

Research Groups and Centers

- the Collective Dynamics Group at the Gulbenkian Institute of Science in Portugal, based in Oeiras;
- the Computational Epidemiology and Public Health Laboratory at ISI Foundation in Italy, based in Turin;
- the Mathematical Modeling Group at the London School of Hygiene and Tropical Medicine in the UK, based in London;

Public Health Institutions

- the Public Health Agency of Sweden, based in Stockholm;
- the National Institute of Health and Medical Research and University Pierre et Marie Curie and the National Institute for Public Health Surveillance in France, based in Paris (this is a peculiar case in that the coordinating team is based at research and public health institutions, and it is also responsible for GP surveillance in the country)

Private Companies

- the Science in Action Company in The Netherlands, based in Amsterdam.

Name and logos for each national platform are shown here below (Figure A1), followed by the dates of the 2011/2012 surveillance season (Table A1).

Country	Network	Logo
Netherlands	deGroteGriepMeting	deGroteGriepMeting.nl Het virus in kaart gebracht voor Nederland en België
Belgium	deGroteGriepMeting	deGroteGriepMeting.nl Het virus in kaart gebracht voor Nederland en België
Portugal	Gripenet	gripenet
Italy	Influweb	INFLUWEB
United Kingdom	Flusurvey	flusurvey
Sweden	Influenzaskoll	www.influensakoll.se Kartlägger förekomst och spridning av influensa i Sverige
France	Grippenet.fr	grippenet.fr

Figure A1. Names and logos of national online surveillance networks participating to the 2011/2012 influenza season.

Table A1. Start and end date of the Influenzanet data collection campaign in the 2011/2012 season.

Influenzanet country	Surveillance start date	Surveillance end date
Belgium (BE)	November 1, 2011	April 30, 2012
France (FR)*	January 25, 2012	April 29, 2012
Italy (IT)	November 18, 2011	April 13, 2012
The Netherlands (NL)	November 1, 2011	April 30, 2012
Portugal (PT)	November 23, 2011	May 21, 2012
Sweden (SE)*	November 17, 2011	May 21, 2012
United Kingdom (UK)	November 10, 2011	April 4, 2012

* first season

2. Analysis of commuting data

2.1 Adjustment of census commuting data

To account for possible discrepancies in the geographic distributions of Influenzanet population with respect to national data, the weight obtained from national commuting sources was corrected for biases in the population distribution by applying the rescaling with the ratio $\frac{N_O^{IN}}{N_O^{GP}}$

between Influenzanet population (IN) and the general population (GP) of the region of origin (N_O^{IN} and N_O^{GP}, respectively). This allowed a direct comparison of the weighted census data with the corresponding links obtained in the Influenzanet sample.

2.2 Extraction of the Backbone of the commuting network through the disparity filter

We explored an alternative method to extract the most relevant backbone of the census commuting networks, called disparity filter algorithm [5] that selects the links displaying statistically significant deviations with respect to a null model for the local assignment of weights to links. The null model used to define anomalous fluctuations is based on the null hypothesis that normalized weights are produced by a random assignment from a uniform distribution. Thus the disparity filter method selects the links, which deviate from the null hypothesis with a certain level of statistical significance α. The level of significance was defined for each country in order to extract a portion of the network with the same size of the corresponding Influenzanet network, in terms of number of links. A visual comparison between the obtained backbone and the Influenzanet network is reported in Figure A6.

2.3 Probability of observing a link in the Influenzanet commuting network

The probability of a link in the census commuting networks to be represented in the Influenzanet network is computed as follow. Given the number of commuters w_{OD} along a given OD direction in the census data, the probability that an individual living in O would commute to D is given by $p_{OD} = w_{OD} / N_O^{GP}$. If we take into account the sample N_O^{IN}, i.e. the number of individuals living in region O in the Influenzanet population, and we assume the events to be independent, the probability that a participant of Influenzanet living in O would commute to region D is given by

$$P_{OD} = 1 - (1 - p_{OD})^{N_O^{IN}} \tag{S1}$$

In Figure 5 of the main paper, we rank links in descending order according to their value of probability and we compare the median rank of the links represented in Influenzanet with the same value for a random sample.

2.4 Probability distribution of the commuter fluxes in the census data

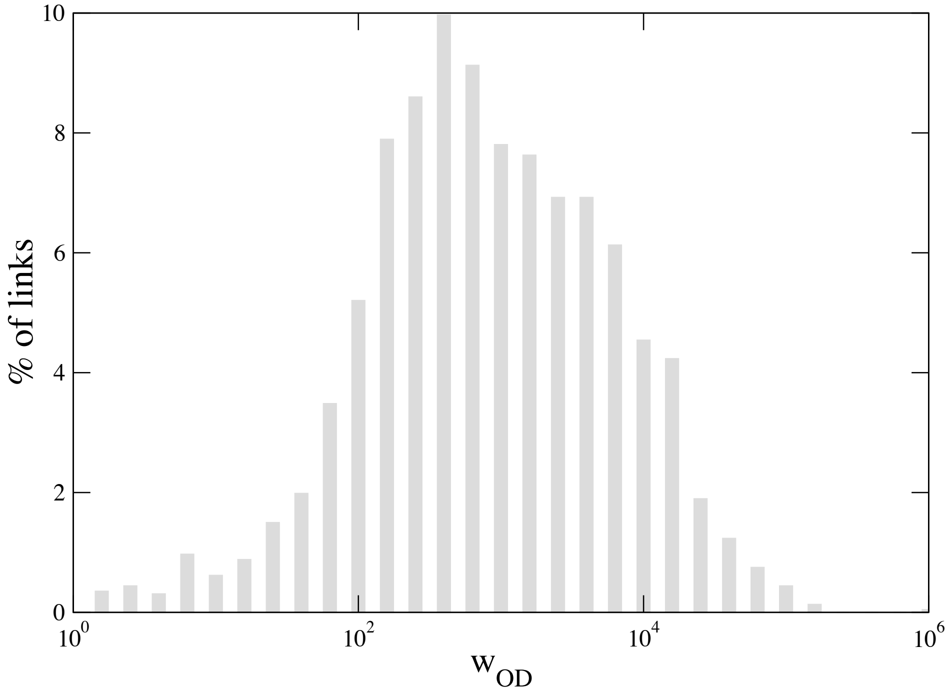

Figure A2. Probability distribution of the commuter fluxes, w_{OD}, in the census data.

3. Supporting results

3.1 Geographical characteristics

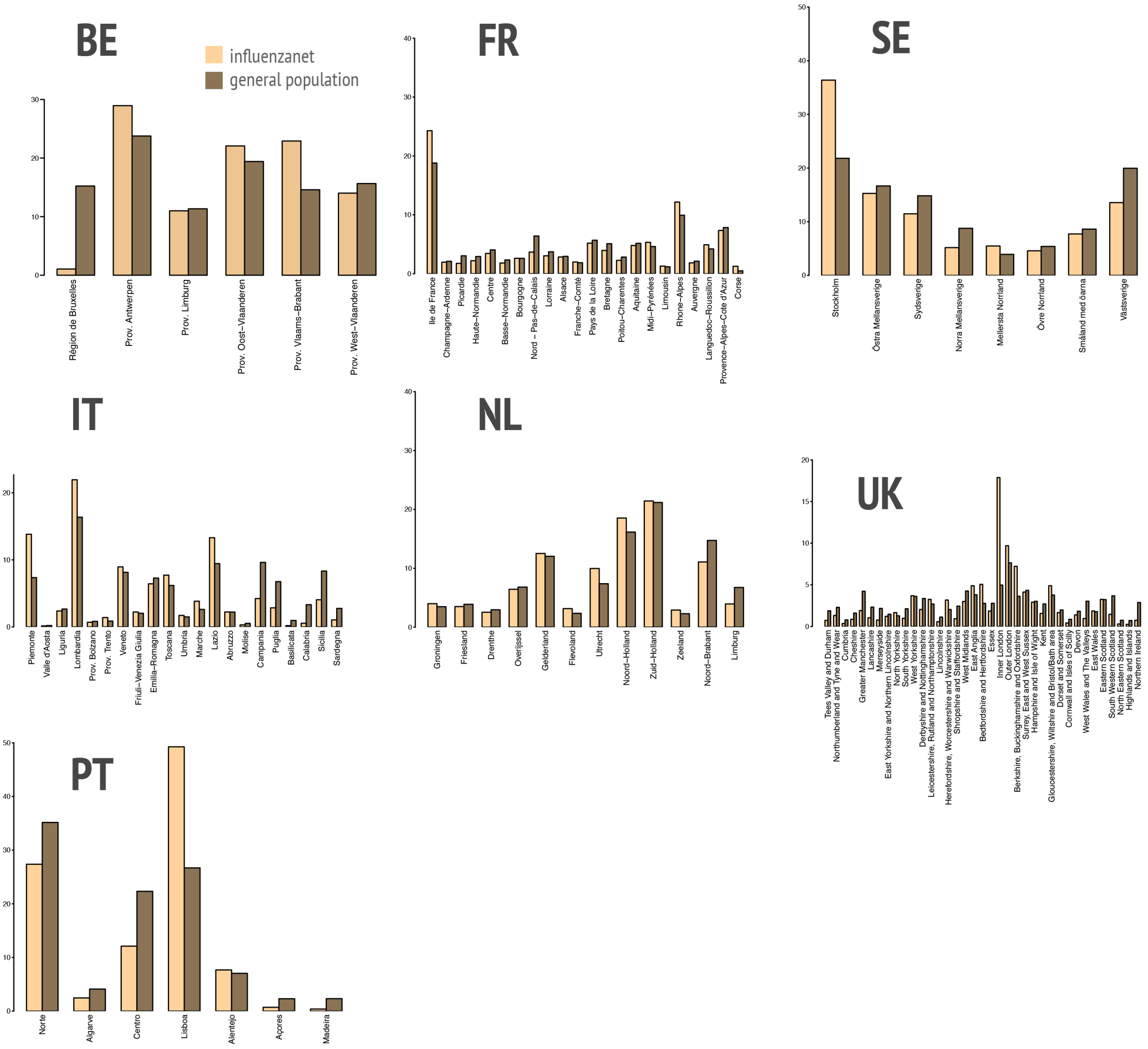

Figure A3. Geographic distribution of Influenzanet population at NUTS2 level and comparison with national data.

3.2 Demographic characteristics

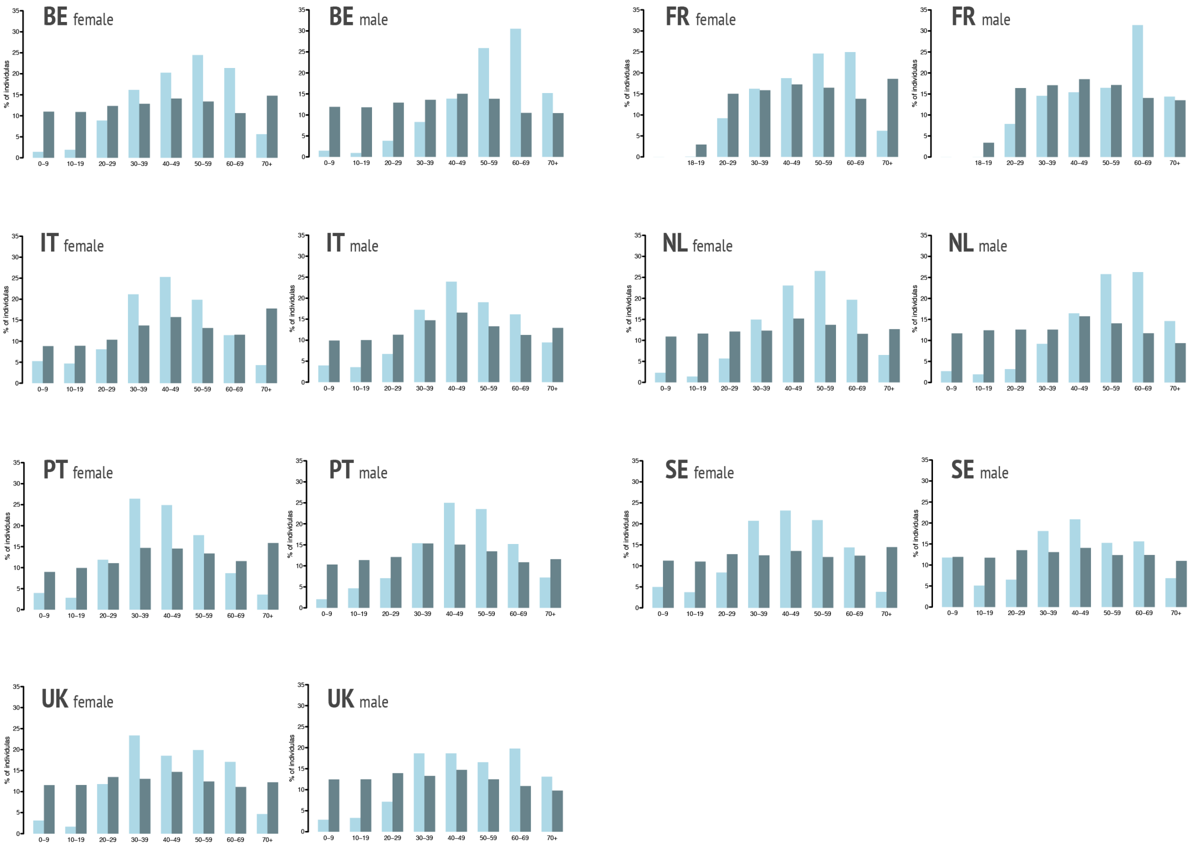

Figure A4. Comparison of Influenzanet population with national data by age and gender.

Table A2. Comparison of Influenzanet population with national data in the [60-64]y and [65-69]y age classes (all $p < 10^{-4}$, except PT for the [65-69]y class for which $p = 0.23$)

Influenzanet Country	Influenzanet [60-64]y, %	General population [60-64]y, %	Influenzanet [65-69]y, %	General population [65-69]y, %
BE	14	5.9	12	4.7
FR	13	6.3	14	4.5
IT	9	6.1	6	5.2
NL	12	6.4	10	5.2
PT	7	6.0	5	5.2
SE	8	6.3	7	6.1
UK	10	5.8	8	5.1

3.3 Mobility

Table A3. Number of links in the Influenza commuting networks at the level of NUTS2 regions and comparison with corresponding census data.

Influenzanet country	Influenzanet # commuting links	General population # commuting links
BE	19	25
FR	27	462
IT	16	152
NL	85	114
PT	9	42
SE	10	56
UK	82	1332

Table A4. Comparison between the Influenzanet commuting networks and the backbones of the corresponding census commuting network.

Influenzanet country	Jaccard Index between Influenzanet network and the backbone of the census commuting network
BE	0.85
FR	0.20
IT	0.23
NL	0.79
PT	0.29
SE	0.18
UK	0.31

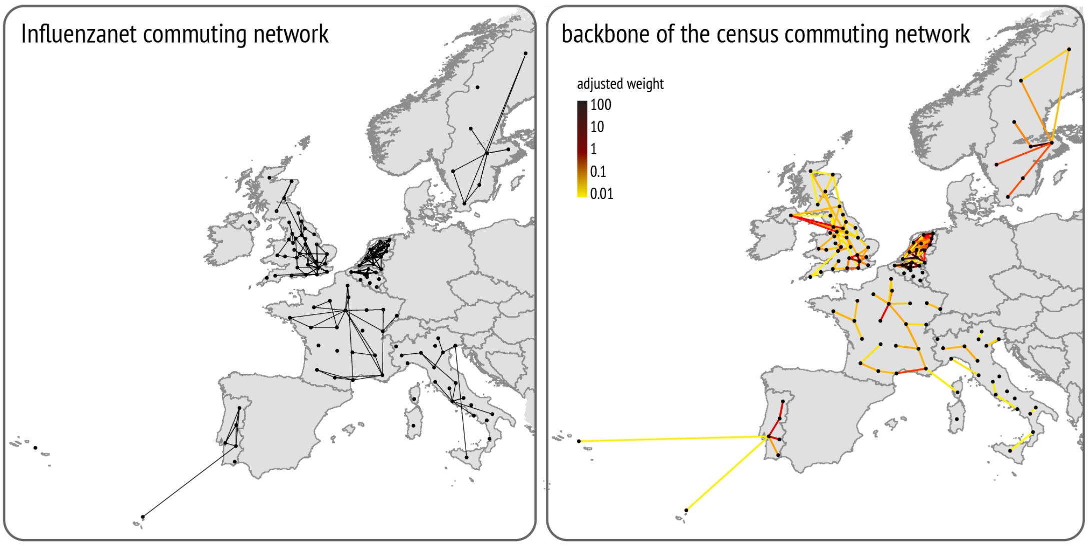

Figure A5. Comparison between the Influenzanet commuting network (left) and the backbone of the census commuting network extracted with the disparity filter algorithm (right). The color code associated to the links in the census commuting network is proportional to the adjusted weight (from the yellow to dark-red). Both networks are directed, arrows are omitted for the sake of visualization.

3.4 Health

Table A5. Smoking prevalence by gender in the population of Influenzanet ≥15 years old and comparison with corresponding national statistics.

Gender	Country	Influenzanet % (95% CI)	General population %	p-value
Female	NE	12.4 (11.5 – 13.3)	19	$< 10^{-6}$
	BE	9.5 (8.1 – 11.0)	18	$< 10^{-6}$
	PT	12.5 (9.7 – 15.8)	11	0.34
	IT	14.3 (11.6 – 17.6)	17	0.11
	UK	7.8 (6.0 – 10.0)	21	$< 10^{-6}$
	SE	10.5 (8.4 – 13.1)	15	$< 10^{-2}$
	FR	18.8 (16.4 – 21.5)	21	0.11
Male	NE	15.3 (14.3 – 16.3)	23	$< 10^{-6}$
	BE	13.9 (12.3 – 15.8)	24	$< 10^{-6}$
	PT	15.2 (12.0 – 19.1)	27	$< 10^{-6}$
	IT	17.9 (14.7 – 21.5)	30	$< 10^{-6}$
	UK	10.1 (8.1 – 12.5)	22	$< 10^{-6}$
	SE	7.7 (5.8 – 10.1)	13	$< 10^{-6}$
	FR	24.8 (21.8 – 28.1)	26	0.48

Table A6. Vaccination coverage for the 2010/2011 season in the 65+ class of individuals in Influenzanet population and comparison with national statistics.

Influenzanet Country	Influenzanet	General population	p value
	% (95% CI)	%	
FR	63 (59 – 67)	54	$< 10^{-4}$
IT	35 (29 – 41)	62	$< 10^{-4}$
NL	80 (78 – 82)	77	$< 10^{-4}$
PT	62 (54 – 69)	40	$< 10^{-4}$
SE	65 (69 – 70)	44	$< 10^{-4}$
UK	75 (69 – 80)	73	0.55

3.5 Following seasons

In this subsection we analyze Influenzanet data for the following two influenza seasons, 2012/2013 and 2013/2014, to test the robustness of the representativeness results presented in the main paper and obtained for the 2011/2012 influenza season.

The analysis on the gender representativeness shows that results are robust across seasons (Table A7).

Table A7. Comparison of Influenzanet population with national data by gender.

Influenzanet Country	Influenzanet	General population	p value
	% males	% males	
**2012/2013 season**			
BE	54.2	49.2	$< 10^{-4}$
FR	38.5	48.4	$< 10^{-4}$
IT	58.3	48.4	$< 10^{-4}$
NL	42.3	49.5	$< 10^{-4}$
PT	47.3	47.6	0.92
UK	37.0	49.2	$< 10^{-4}$
**2013/2014 season**			
BE	54.5	49.2	$< 10^{-4}$
FR	39.4	48.4	$< 10^{-4}$
IT	59.2	48.4	$< 10^{-4}$
NL	42.7	49.5	$< 10^{-4}$
PT	47.6	47.6	0.99
UK	37.5	49.2	$< 10^{-4}$

* SE implemented a different standard for participatory surveillance, so it is not included in this sensitivity analysis

The results on the breakdown by age classes (10y) and gender confirm the same qualitative trend observed for the 2011/2012 season. Figures A6 and A7 reports the results for 4 countries for the sake of simplicity. Few differences can be noted: for example, in the 2013/2014 season, the increase of participation in the school-age children in the UK, due to the implementation of school communication campaigns and activities for Flusurvey [7].

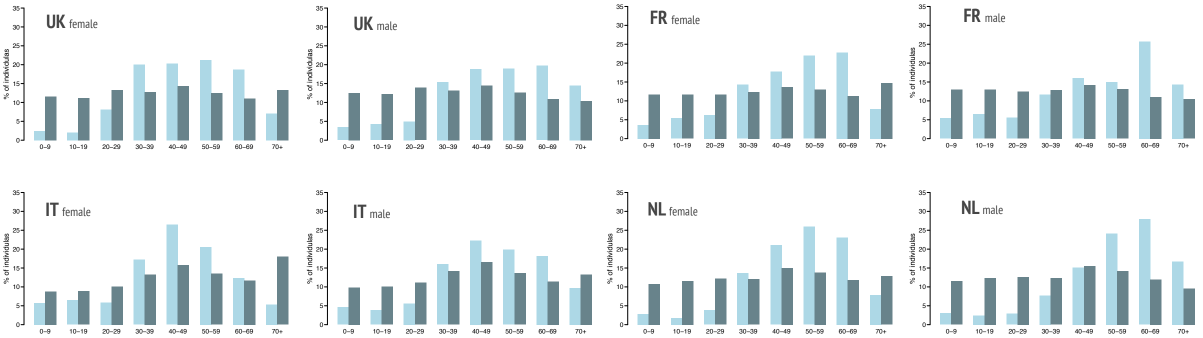

Figure A6. Comparison of Influenzanet population with national data by age and gender, for the season 2012/2013.

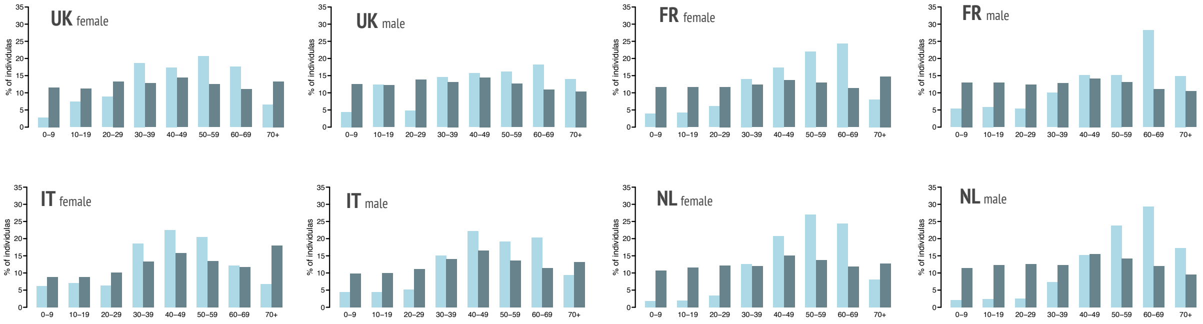

Figure A7. Comparison of Influenzanet population with national data by age and gender, for the season 2013/2014.

Finally, we report on the results regarding the health indicators, such as asthma (Table A8) and diabetes (Table A9) incidence, and the vaccination coverage (Table A10). Fewer countries than the total are listed in the Tables, due to the lack of data for the general population for the years corresponding to the seasons under study for the missing countries. Results are qualitatively the same as those obtained for the 2011/2012 season, except for the Influenzanet vaccination coverage in France that is found to be representative of the coverage in the general population in the seasons 2012/2013 and 2013/2014, differently from what observed in the previous season.

Table A8. Asthma incidence: comparison of Influenzanet population with national data.

Influenzanet Country	Influenzanet %	General population %	p value
**2012/2013 season**			
FR	6.6	6.5	0.71

IT	3.9	6.1	$<10^{-4}$
UK	6.0	9.5	$<10^{-4}$
**2013/2014 season**			
FR	6.4	6.5	0.81
IT	3.8	5.9	$<10^{-4}$
UK	6.1	9.6	$<10^{-4}$

Table A9. Diabetes incidence: comparison of Influenzanet population with national data.

Influenzanet Country	Influenzanet	General population	p value
	%	%	
**2012/2013 season**			
FR	2.6	2.7	0.84
IT	2.2	5.5	$<10^{-4}$
UK	2.9	4.6	$<10^{-4}$
**2013/2014 season**			
FR	2.6	2.7	0.84
IT	2.2	5.4	$<10^{-4}$
UK	2.8	4.8	$<10^{-4}$

Table A10. Vaccination coverage in the 65+ age class: comparison of Influenzanet population with national data.

Influenzanet Country	Influenzanet	General population	p value
	%	%	
**2012/2013 season**			
FR	59	53	0.59
IT	46	54	0.46
UK	75	74	0.75
**2013/2014 season**			
FR	60	53	0.61
IT	48	54	0.48
UK	79	73	0.79

References

1. Inserm (2013) Institut national de la santé et de la recherche médicale (updated April 2013). Available: http://www.inserm.fr/. Accessed 2013 April 25.
2. UPMC (2013) Université Pierre et Marie Curie(updated April 2013). Available: http://www.upmc.fr/. Accessed 2013 April 25.
3. Flahault A, Blanchon T, Dorleans Y, Toubiana L, Vibert JF, et al. (2006) Virtual surveillance of communicable diseases: a 20-year experience in France. Stat Methods Med Res 15: 413-421.
4. InVS (2013) Institut de veille sanitaire(updated April 2013). Available: http://www.invs.sante.fr/. Accessed 2013 April 25.
5. Serrano MA, Boguna M, Vespignani A. Extracting the multiscale backbone of complex weighted networks. Proc Natl Acad Sci U S A. 2009 Apr 21;106(16):6483-8.
6. D Paolotti, A Carnahan, V Colizza, K Eames, J Edmunds, G Gomes, C Koppeschaar, M Rehn, R Smallenburg, C Turbelin, S Van Noort, A Vespignani. Web-based participatory surveillance of infectious diseases: the Influenzanet participatory surveillance experience. *Clinical Microbiology and Infection* 20:17-21 (2014).
7. British Science Association. Flusurvey School Project. http://www.britishscienceassociation.org/flusurvey-school-project
