# Supplementary material for: The representativeness of a European multi-center network for influenza-like-illness participatory surveillance
Source: BMC Public Health. 2014 Sep 20;14:984. doi: 10.1186/1471-2458-14-984 (PMC4192744; doi:10.1186/1471-2458-14-984)
Supplement: Supplementary file 2 — Additional file 2: The file contains the Influenzanet intake survey in its English version. (PDF 158 KB) [file 12889_2014_7121_MOESM2_ESM.pdf]

Additional File 2

The representativeness of a European multi-center network for influenza-like-illness participatory surveillance

Pietro Cantarelli[1,2,3], Marion Debin[1,2], Clément Turbelin[1,2], Chiara Poletto[1,2], Thierry Blanchon[1,2], Alessandra Falchi[1,2], Thomas Hanslik[1,2,4], Isabelle Bonmarin[5], Daniel Levy-Bruhl[5], Alessandra Micheletti[3], Daniela Paolotti[6], Alessandro Vespignani[6,7,8], John Edmunds[9], Ken Eames[9], Ronald Smallenburg[10], Carl Koppeschaar[10], Ana O. Franco[11], Vitor Faustino[11], AnnaSara Carnahan[12], Moa Rehn[12], Vittoria Colizza[1,2,6*]

1) INSERM, UMR-S 1136, Institut Pierre Louis d'Epidémiologie et de Santé Publique, Paris, France
2) Sorbonne Universités, UPMC Univ Paris 06, UMR-S 1136, Institut Pierre Louis d'Epidémiologie et de Santé Publique, Paris, France
3) Università degli Studi di Milano, Milan, Italy
4) Assistance Publique Hopitaux de Paris, Service de Medecine Interne, Hopital Ambroise Pare, Boulogne Billancourt, France
5) Department of infectious diseases. Institut de Veille Sanitaire (InVS), St Maurice Cedex, 94415, France
6) Institute for Scientific Interchange (ISI), Turin, Italy
7) Laboratory for the Modeling of Biological and Socio-technical Systems Northeastern University, Boston, USA
8) Institute for Quantitative Social Sciences at Harvard University, Cambridge, USA
9) London School of Hygiene and Tropical Medicine, Great Britain
10) Aquisto-Inter BV, Amsterdam, The Netherlands
11) Instituto Gulbenkian de Ciência, Oeiras, Portugal
12) Public Health Agency of Sweden, Sweden

Influenzanet intake survey (English version)

Mandatory questions are followed by a *.
Where the options is “pick only one” (e.g. Intake Q1) we used filled bullet points; where it is “pick all that apply” (e.g. Intake Q5) we used open bullet points.

Intake Q0
For whom are you filling this survey in?*

- Myself
- A member of my household
- Someone else

Intake Q0b
I hereby certify that I have explained to this people the content and implications of this study, and obtained his free consent to participate. This people gave me the authorisation to answer to his questionnaires.*

- Yes
- No

If « No », it is not possible to fill in the questionnaire, or to answer to other questionnaires. If « Yes », the following text appears : « Please answer to all these questions as if you were this person ».

Intake Q1
What is your gender?*

- Male
- Female

Intake Q2
What is your date of birth (month and year)?*
If the date of birth indicates that the participant is under 18 , it is not possible to fill in the questionnaire, or to answer other questionnaires.

Intake Q3
What is your home postal code?*
This information is not registered in the database, and only allows answering easily to the next question.

Intake Q3b *[only available in the French version]*
What is your home municipality?*
Drop-down list thanks to the answer of the question Q3.

Intake Q4
What is your main activity?*

- Paid employment, full-time
- Paid employment, part-time
- Self-employed (businessman, farmer, tradesman, etc)
- Attending school/college/university
- Home-maker (e.g. housewife)
- Unemployed
- Long-term sick-leave or parental leave
- Retired
- Other

Intake Q4b (if Yes to Q4)

What is your school/college/workplace postal code (where you spend the majority of your working/studying time)?

- XXXX
- I don't know/can't remember
- Not applicable (e.g. don't have a fixed workplace)

This information is not registered in the database, and only allows answering easily to the next question.

Intake Q4c *[only available in the French version]*
What is your school/college/workplace municipality?
Drop-down list thanks to the answer of the question Q3.

Intake Q4d (if "Yes, Paid employment full time", or "Yes, paid employment part time" to Q4)
Which of the following descriptions most closely matches with your main occupation?

- Professional (e.g. manager, doctor, teacher, nurse, engineer)
- Office work (e.g. admin, finance assistant, receptionist, etc)
- Retail, sales, catering and hospitality and leisure (e.g. shop assistant, waiter, bar-staff, gym instructor etc)
- Skilled manual worker (e.g. mechanic, electrician, technician)
- Other manual work (e.g. cleaning, security, driver)
- Other

Intake Q4e (to be asked for those aged 16 and over)
What is the highest level of formal education qualification that you have? If you are still in education, then please tick this box, with the appropriate highest level that you have already achieved.

- I have no formal qualifications
- GCSE's, O'levels, CSEs or equivalent
- A-Levels or equivalent (e.g. Highers, NVQ Level3, BTEC)
- Bachelors Degree (BA, BSc) or equivalent (e.g. HND, NVQ Level 4)
- Higher Degree or equivalent (e.g. Masters Degree, PGCE, PhD, Medical Doctorate, Advanced Professional Awards)
- I am still in education

Intake Q5
Do you have contact with any of the following during the course of a typical day?* (Select all options that apply, if any)

- More than 10 children or teenagers over the course of the day
- More than 10 people aged over 65 over the course of day
- Patients
- Groups of people (more than 10 individuals at any one time)
- None of the above

Intake Q6
INCLUDING YOU, how many people in each of the following age groups live in your household?*
Drop down menus for each of:
I live alone
0-4 years
5-18 years
19-44 years
45-64 years
65+ years

Intake Q6b (If any in household are aged 0-4, including participant)
How many of the children aged 0-4 in your household go to school or day-care?

Intake Q6c (if answer to question Q6b is not 0)
Where do those children go during the day?

- School
- Nursery Assistant
- Joint custody
- Nursery
- Kindergarten
- Family day-care home
- Day-care

Intake Q7
What is your main means of transportation?*

- Walking
- Bike
- Motorbike/scooter
- Car
- Public transportation (bus, train, tube, etc.)
- Other

Intake Q7b
On a normal day, how much time do you spend on public transport (bus, train, tube, etc)?*

- No time at all
- 0-30 minutes
- 30 minutes – 1.5 hours
- 1.5 hours – 4 hours
- Over 4 hours

Intake Q8
How often do you have common colds or flu-like diseases?

- Never
- Once or twice a year
- Between 3 and 5 times a year
- Between 6 and 10 times a year
- More than 10 times a year
- I don't know

Intake Q9a *[only available in the French version]*
Did you receive a vaccination voucher for influenza, this year? *

- Yes
- No
- I don't know/can't remember

Intake Q9b *[only available in the French version]*
What is your height (in centimeters)?

Intake Q9c *[only available in the French version]*
What is your weight (in kilograms)?

Intake Q10
Did you receive a flu vaccine this autumn/winter season (since last September)?

- Yes [go to questions Q10b and Q10c]
- No [go to question Q10d]
- I don't know/can't remember

Intake Q10b (if “Yes” to Intake Q10)
When were you vaccinated against flu this season (2011-2012)?

- XX/XX/XXXX
- I don’t know/can’t remember

Intake Q10c (if “Yes” to Intake Q10)
What were your reasons for getting a seasonal influenza vaccination this year? (Select all options that apply)

- I received a voucher at home *[only available in the French version]*
- I belong to a risk group, for which vaccination is recommended (pregnant women, people over 65, chronic disease condition, contact with children at risk and under 6 months, living in a long term care institution, obese, etc.)
- Vaccination decreases my risk of getting influenza
- Vaccination decreases the risk of spreading influenza to others
- My doctor recommended it
- It was recommended in my workplace/school
- The vaccine was readily available and vaccine administration was convenient
- The vaccine was free
- I don’t want to miss work/school
- I always get the vaccine
- Other reason(s)

Intake Q10d (if “No” to Intake Q10, follow-up question)
What were your reasons for NOT getting a seasonal influenza vaccination this year? (Select all options that apply)

- I am planning to be vaccinated, but haven’t been yet
- I haven’t been offered the vaccine
- I don't belong to a risk group for which the vaccination is recommended (pregnant women, people over 65, chronic disease condition, contact with children at risk and under 6 months, living in a long term care institution, obese, etc.)
- It is better to build your own natural immunity against influenza
- I doubt that the influenza vaccine is effective
- Influenza is a minor illness
- I don’t think that I am likely to get influenza
- I believe that influenza vaccine can cause influenza
- I am worried that the vaccine is not safe or will cause illness or other adverse events
- I don’t like having vaccinations
- The vaccine is not readily available to me
- The vaccine is not free of charge
- No particular reason
- Although my doctor recommended a vaccine, I did not get one
- Other reason(s)

Intake Q11
Have you received a flu vaccine during the last autumn/winter season (i.e. 2010-2011)?*

- Yes
- No
- I don’t know/can’t remember

Intake Q12
Do you take regular medication for any of the following medical conditions?* (Select all options that apply)

- No, I do not have any of these diseases
- Asthma
- Diabetes
- Lung disorder (COPD, emphysema, …)
- Heart disorder
- Kidney disorder

- An immunocompromising condition (e.g. splenectomy, organ transplant, acquired immune deficiency, cancer treatment)

Intake Q13 (only asked of women between ages 15 and 50)
Are you currently pregnant?

- Yes [go to question Q13b]
- No [go to question Q14]
- Don't know/would rather not answer

Intake Q13b: (if "Yes" to Intake Q12)
Which trimester of the pregnancy are you in?

- First trimester (week 1-12)
- Second trimester (week 13-28)
- Third trimester (week 29-delivery)
- Don't know/would rather not answer

Intake Q14
Do you smoke tobacco?*

- No, I never smoked
- No, I stopped smoking more than one year ago
- No, I stopped smoking less than one year ago
- Yes, occasionally
- Yes, daily, fewer than 10 times a day
- Yes, daily, 10 or more times a day
- Don't know/would rather not answer

Intake Q15
Do you have one of the following allergies that can cause respiratory symptoms?* (Select all options that apply)

- Hay fever
- Allergy against house dust mite
- Allergy against domestic animals or pets
- Other allergies that cause respiratory symptoms (e.g. sneezing, runny eyes)
- I do not have an allergy that causes respiratory symptoms

Intake Q16
Do you follow a special diet? (Select all options that apply)

- No special diet
- Vegetarian
- Veganism
- Low-calorie
- Other

Intake Q17
Do you have pets at home? (Select all options that apply)

- No
- Yes, one or more dogs
- Yes, one or more cats
- Yes, one or more birds
- Yes, one or more other animals

Intake Q18
Where did you hear about the Influenzanet project for the first time? *["Influenzanet project" was substituted in each country with the national online surveillance network]*

- On radio or television
- In the newspaper or in a magazine

- Via an Internet site (search engine or link)
- By the poster of the project
- Via family or friends
- Via school or work
- Via an healthcare professional
- A member of my family/friends work on this project
